# Analyzing the Systems Biology Effects of COVID-19 mRNA Vaccines to Assess Their Safety and Putative Side Effects

**DOI:** 10.3390/pathogens11070743

**Published:** 2022-06-29

**Authors:** Rima Hajjo, Dima A. Sabbah, Alexander Tropsha

**Affiliations:** 1 Department of Pharmacy, Faculty of Pharmacy, Al-Zaytoonah University of Jordan, P.O. Box 130, Amman 11733, Jordan; dima.sabbah@zuj.edu.jo; 2 Laboratory for Molecular Modeling, Division of Chemical Biology and Medicinal Chemistry, Eshelman School of Pharmacy, The University of North Carolina at Chapel Hill, Chapel Hill, NC 27599, USA; alex_tropsha@unc.edu; 3 Jordan CDC, Zahran St 137, Amman 11181, Jordan

**Keywords:** COVID-19, mRNA vaccine, informatics workflow, SARS-CoV-2, systems biology, vaccine adverse events, VAERS

## Abstract

COVID-19 vaccines have been instrumental tools in reducing the impact of SARS-CoV-2 infections around the world by preventing 80% to 90% of hospitalizations and deaths from reinfection, in addition to preventing 40% to 65% of symptomatic illnesses. However, the simultaneous large-scale vaccination of the global population will indubitably unveil heterogeneity in immune responses as well as in the propensity to developing post-vaccine adverse events, especially in vulnerable individuals. Herein, we applied a systems biology workflow, integrating vaccine transcriptional signatures with chemogenomics, to study the pharmacological effects of mRNA vaccines. First, we derived transcriptional signatures and predicted their biological effects using pathway enrichment and network approaches. Second, we queried the Connectivity Map (CMap) to prioritize adverse events hypotheses. Finally, we accepted higher-confidence hypotheses that have been predicted by independent approaches. Our results reveal that the mRNA-based BNT162b2 vaccine affects immune response pathways related to interferon and cytokine signaling, which should lead to vaccine success, but may also result in some adverse events. Our results emphasize the effects of BNT162b2 on calcium homeostasis, which could be contributing to some frequently encountered adverse events related to mRNA vaccines. Notably, cardiac side effects were signaled in the CMap query results. In summary, our approach has identified mechanisms underlying both the expected protective effects of vaccination as well as possible post-vaccine adverse effects. Our study illustrates the power of systems biology approaches in improving our understanding of the comprehensive biological response to vaccination against COVID-19.

## 1. Introduction

COVID-19 vaccines have been instrumental tools in reducing the impact of SARS-CoV-2 infections around the world. Vaccines reduced the impact of SARS-CoV-2 infections around the world by preventing 80% to 90% of hospitalizations and deaths from reinfection, in addition to preventing 40% to 65% of symptomatic illnesses [1]. All vaccines have protected individuals from severe disease, hospitalizations and death, especially during the first six months of being fully-vaccinated [2,3,4,5,6,7,8,9,10,11,12]. Vaccines have also interrupted viral transmission chains, prevented outbreaks in largely-vaccinated communalities, and reduced the need for lockdowns and very strict pandemic control measures [13]. The vaccines that are currently approved/authorized for emergency use by the FDA or WHO were prepared using three technologies: (1) mRNA vaccines, (2) viral vector vaccines, and (3) inactivated vaccines.

The mRNA vaccines have been among the best-performing vaccines, showing the greatest efficacy in preventing symptomatic COVID-19 disease in clinical trials with efficacies reaching 95% and 94.5% for Pfizer-BioNTech’s BNT162b2 (FDA-approved) and Moderna’s mRNA-1273 (authorized for emergency use by the FDA) vaccines, respectively [14]. In addition to their high potency, these vaccines were easy to develop and safer to produce. Additionally, a newer mRNA vaccine (CVnCoV) is being developed by CureVac, an international biopharmaceutical company with headquarters in Tübingen, Germany. CureVac is currently collaborating with GlaxoSmithKline (GSK) to finalize phase 3 clinical studies and get the vaccine approved in 2022. Recent clinical studies showed that CVnCoV is safe and possesses adequate immunogenicity resulting in a dose-dependent neutralizing antibody response [15]. At a dose of 12 µg, CureVac’s vaccine induces neutralizing antibody titers comparable to those detected in the serum of people previously infected with SARS-CoV-2 [15,16]. We summarize all findings about the three abovementioned advanced mRNA vaccines in Table 1.

Unlike older conventional vaccines, the mRNA vaccine platform is cell-free, where the antigen is encoded as messenger RNA using an in vitro transcriptional process and then encapsulated within lipid nanoparticles (LNPs) which facilitate the cytoplasmic delivery of the immunogen through a fusion-based mechanism [8,37,43,44,45,46,47,48,49,50,51,52,53,54,55]. In fact, the LNP content also prevents mRNA degradation and improves its cellular entry. All mRNA vaccines described here deploy ionizable cationic lipids in their NLPs [27,56], as shown in Table 1. Studies have indicated that the mRNA molecule along with the ionizable cationic lipid content and water are all located within the LNP core, while the neutral lipids are always present on the external surface surrounding the mRNA content [21].

Despite being essential tools in the fight against COVID-19, mRNA vaccines are still instigating fear related to the lack of information regarding their short-term and long-term side effects, especially after a putative link between mRNA vaccines and post-vaccine myocarditis in male adolescents and young male adults was identified [57,58,59,60]. Herein, we are trying to shed light on the systems biology effects of COVID-19 mRNA vaccines to assess their safety and putative adverse events based on transcriptomics data for Pfizer-BioNTech’s BNT162b2 in vaccinated individuals. No such data were publicly available for Moderna’s mRNA-1273 at the time we conducted these analyses. Our results, however, can be generalized to other mRNA vaccines based on evidence from the biomedical literature [61,62,63].

We applied a systems biology workflow to shed light on post-mRNA biological effects and provide some answers for the most frequently asked questions about vaccine safety being raised recently by both experts and the general public. Our approach evaluates the chemogenomic effects of vaccines and vaccine compositions at the systems biology level to generate testable hypotheses about the effects of vaccines on biological systems. This approach has been validated and used successfully to study the systems biology effects of small molecule drugs and non-COVID-19 vaccines by our group [64,65,66,67] and by others [68,69,70,71].

## 2. Results

**Figure 1:**
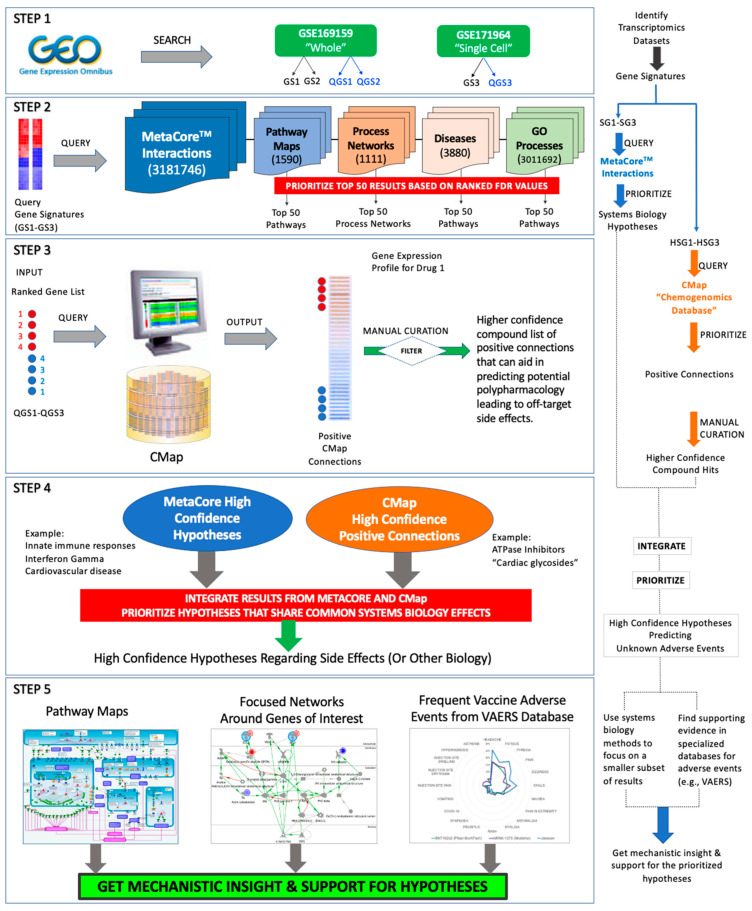
Systems biology workflow to study potential post-vaccine side effects.

We applied a systems biology workflow (Figure 1) that supports enrichment analysis and prioritization of small molecule drugs that mimic the action of a COVID-19 mRNA vaccine (BNT162b2). This workflow was first described by Hajjo et al. [72] and has been applied to many drug and vaccine discovery projects [73,74,75,76].

### 2.1. Deriving Transcriptional Signatures for COVID-19 mRNA Vaccines

Our analysis of transcriptional raw data extracted from GSE169159 indicated that gene expression alterations from baseline were more prominent on day 22 (i.e., on the day after receiving the second dose). Other time points did not pass the thresholds for selecting DEGs (i.e., log_2_ fold change (log_2_FC) of ≥2 or ≤−2, and false discovery rate (FDR) ≤ 0.05). Therefore, we relied on differential gene expressions on day 22 for systems biology analysis of BNT162b2 effects. High-throughput sequencing data from GSE169159 and single-cell CITE-seq from GSE171964 were used to derive three transcriptomics signatures for BNT162b2 vaccine: (1) gene signature 1 (GS1) consisting of 1853 differentially expressed genes DEGs (884 upregulated, 969 downregulated) with false discovery rate (FDR) ≤ 0.05 and a log_2_ fold change (log_2_FC) ≥2 or ≤−2; (2) GS2 consisting of 210 DEGs (138 upregulated, 72 downregulated) with FDR ≤ 0.05 and a log_2_ fold change (log_2_FC) ≥ 4 or ≤−4; (3) GS3 consisting of 155 DEGs (27 upregulated,128 downregulated) from single-cell transcriptomics data comparing day 22, i.e., the day after receiving the booster dose, with baseline data on day 1, i.e., on the same day of receiving the priming vaccine dose just before receiving the vaccine, across all processed cell lines, with FDR ≤ 0.05 and log_2_FC ≥ 2 or ≤−2. All details about the gene expression data sets and derived transcriptional gene signatures are found in the Methods sections and in Supplementary Table S1.

**Figure 2:**
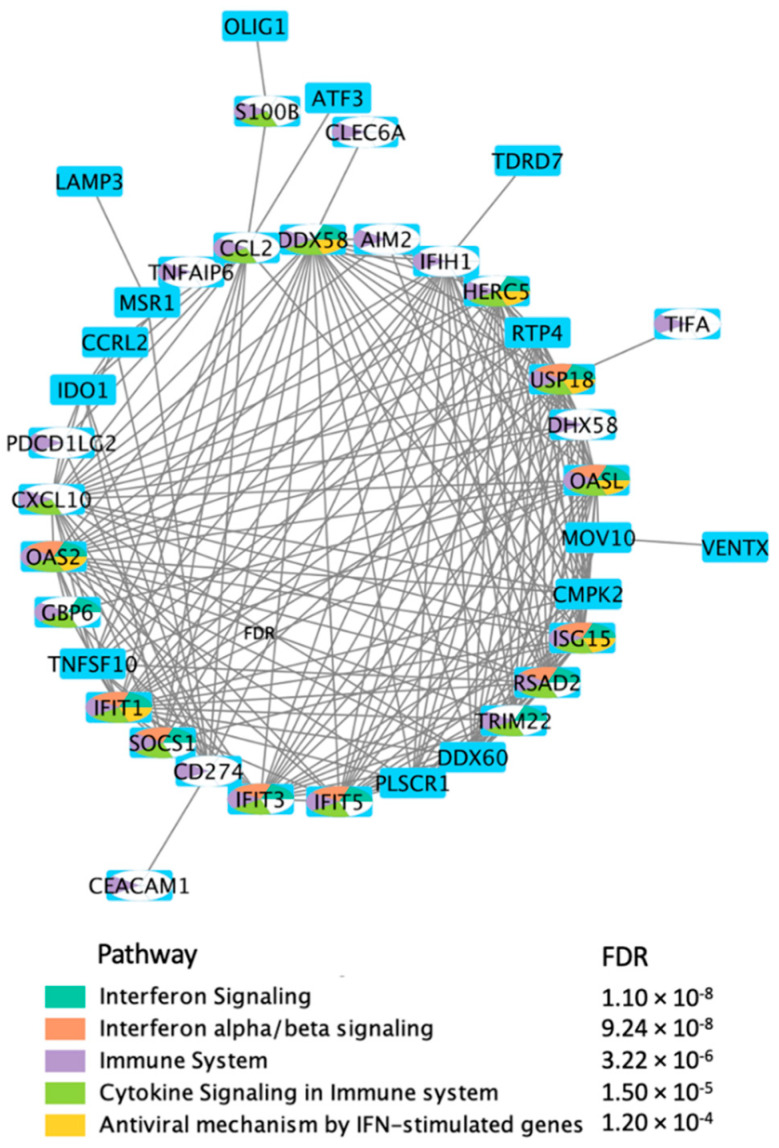
Direct protein–protein interaction (PPI) network using the DEGs, in response to BNT162b2 vaccine on day 22, selected by applying log_2_FC threshold of ±5 and FDR ≤ 0.05.

In order to get a better idea about the biological significance of the DEGs in response to treatment with BNT162b2, we generated direct interactions networks using the 76 DEGs after applying the log_2_FC threshold of ±5 and FDR ≤ 0.05. We also used these DEGs for pathway enrichment analysis according to the description in the Methods section. Our results (Figure 2) indicate that the selected 76 DEGs are involved in various immune processes including: interferon signaling, cytokine signaling, interferon alpha/beta signaling, antiviral mechanisms by interferon-stimulated genes, and interferon gamma (INF-gamma) signaling.

### 2.2. Enrichment Analysis of the Transcriptional Signatures

**Figure 3:**
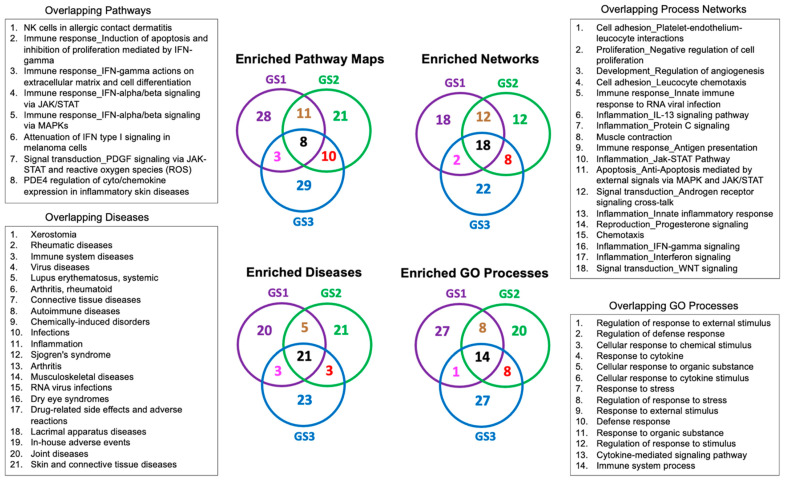
Enrichment results for BNT162b2 vaccine using three transcriptional gene signatures (GS1–GS3).

We used three transcriptional gene signatures’, GS1–GS3, for the BNT162b2 vaccine to study the systems biology effects of mRNA vaccines. Our methods relied on performing rigorous enrichment analyses using the MetaCore^TM^ database [77]. The top 50 most significant enrichment results for the vaccine are provided in Supplementary Table S2. This table shows enrichments in pathway maps, process networks, diseases, and gene ontology (GO) processes. The enrichment results for the vaccine were further filtered by prioritizing the overlapping higher confidence hypotheses shown in the Venn diagrams in Figure 3.

**Figure 4:**
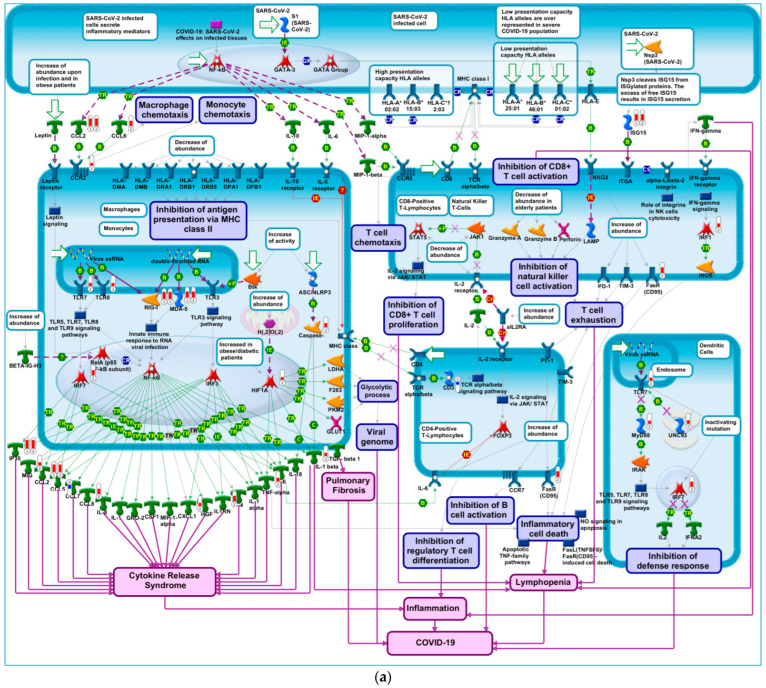

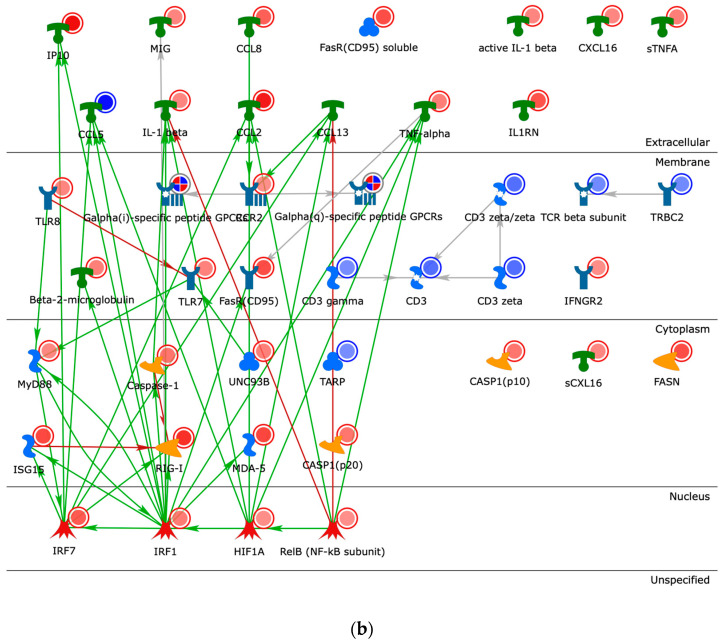
COVID-19 immune dysregulation. (**a**) A node (or object) on the map could be a gene, protein or chemical compound. Query genes from experimental data which intersect with pathway objects are denoted by thermometers. Thermometer 1 represents DEGs in response to treatment with vaccine, applying thresholds of 5 and −5 on log_2_FC and FDR ≤ 0.05, respectively. Thermometer 2 represents DEGs in response to treatment with vaccine, applying thresholds of 2 and −2 on log_2_FC and FDR ≤ 0.05, respectively. (**b**) Direct interactions network map between DEGs enriching COVID-19 immune dysregulation pathways. Connections between network objects on the map are referred to as links (or edges). A link identifies an interaction or a logical relation between two nodes. The type of interaction or relation is reflected by an appropriate symbol placed in the middle of the link. B = binding; IE = influence on expression; TR = transcription regulation; red arrows = inhibition; green arrows = activation; grey arrows = unspecified action; light violet text box = normal process; pink text box = pathological processes; white text box with blue outline = notes; starred network objects = groups or complex processes; red thermometers on pathway map = network object is upregulated by vaccine; blue thermometers on pathway map = network object is downregulated by vaccine. The length of red and blue bars in the thermometers represent log_2_FC values (longer red bars represents larger upregulation of gene expression and longer blue bars represent larger downregulation of gene expression). Red circles on process network = network object is upregulated by vaccine; blue circles on process network = network object is downregulated by vaccine. Darker red circles indicate larger upregulation, darker red circles indicate larger downregulation.

Genes that have greater effects on transcription (e.g., transcription factors), or those that show greater aberrations in response to treatment, are known to exert greater effects on modulating the underlying biology. Therefore, we performed enrichment analyses in pathway maps using multiple lists of DEGs selected by applying different thresholds on log_2_FC values. Thresholds of (8, −8), (7, −7), and (6, −6) did not result in appropriate query gene lists (i.e., consisting of 100–1000 DEGs) for querying MetaCore^TM^ pathway maps. Notably, log_2_FC thresholds of (3, −3), (4, −4) and (5, −5), for both upregulated and downregulated genes, resulted in query genes lists (in the hundreds) with optimal size, and their enrichment results highlighted several important pathway maps (Table 2) including “COVID-19 immune dysregulation”, shown in Figure 4a, and “COVID-19: SARS-CoV-2 effects on infected tissues”. Further analysis of the DEGs that led to the enrichment of “COVID-19 immune dysregulation” was performed by generating a direct interactions network, shown in Figure 4b, using the direct interactions network building algorithm in MetaCore^TM^. Our results show that 69% of network objects corresponding to DEGs, shown in Figure 4a and indicated by means of red and blue thermometers, are connected via high-trust curated interactions. The remaining 31% of network objects corresponding to the DEGs that led to the enrichment of “COVID-19 immune dysregulation” are connected to the larger process network via genes known to affect COVID-19 immune dysregulation, as shown in Figure 4b. It is notable that two genes in this network, IP10 and CCL2, had a log_2_FC exceeding 5. In fact, both genes are linked to thromboinflammation and SARS-CoV-2 infection [78]. Interferon gamma inducible protein-10 (IP10) is a cytokine related to thrombosis and may be a key regulator of the cytokine storm immune repones to SARS-CoV-2 infection. Recent research indicated that the level of IP10 in the serum of critically ill patients was higher than that in severe patients, suggesting that IP10 can be used as a biomarker for COVID-19 disease severity [79]. Additionally, elevated expression of the protein encoded by CCL2 is associated with SARS-CoV-2.

### 2.3. Identifying Transcriptomics Similarities with Small-Molecule Drugs to Aid in Predicting Potential Adverse Events

In order to identify chemical compounds capable of inducing transcriptomics effects similar to those induced by mRNA vaccines, we ranked all DEGs according to their expression levels using log_2_FC values, and prepared three gene lists to query the Connectivity Map database [80]. Each query gene signature (QGS) consisted of about the 50 most upregulated genes and the 50 most downregulated genes in response to treatment with the vaccine. QGS1 was obtained by ranking the DEGs for the whole transcriptomics dataset on day 22 by focusing on all DEGs with *p*-values ≤ 0.05, while QGS2 was obtained by ranking the DEGs from whole transcriptomics data on day 22 by focusing on DEGs with FDR ≤ 0.05. Finally, QGS3 was obtained by ranking the DEGs from single-cell transcriptomics data which had FDR ≤ 0.05. Compound hits that produced similar transcriptional signatures to the mRNA vaccine of BNT162b2 are listed in Table 3. In this study, we wanted to increase the confidence in the computational hypotheses derived from the CMap, so we compared vaccine-compound similarities based on their gene perturbation effects by focusing on subsets of DEGs that have been prioritized using different experiments and ranked by different methods. If query gene lists that share few or no common genes lead to similar lists of compound hits, this increases our confidence that the results are not only statistically robust, but also biologically relevant.

Our results highlighted thirteen high confidence hits (Table 3) which belonged to six pharmacological classes: (1) four ATPase inhibitors (38.46%), (2) three protein synthesis inhibitors (30.76%), (3) one Bruton’s Tyrosine Kinase (BTK) inhibitor (7.69%), (4) one apoptosis stimulant (7.69%), (5) one ribonucleoside reductase inhibitor (7.69%), and (6) one guanylate cyclase activator (7.69%). In fact, all top 12 positive compound connections shown in Table 3 are known modulators of the immune system through their effects on innate immune pathways such as stimulating apoptosis [81] NLRP3 inflammasome pathways.

### 2.4. Building Networks for Drug Targets of CMap Compound Hits to Gain Biological Insights

**Figure 5:**
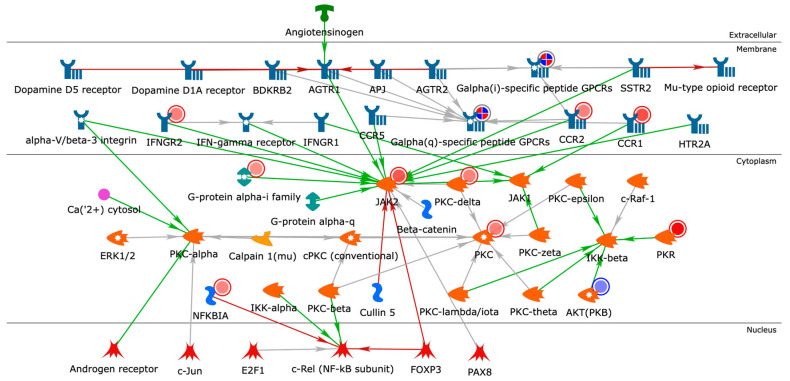
Closest-neighbor network of the identified drug targets for CMap compound hits. Objects/nodes = proteins, transcription factors, compounds, ions, reactions; blue-encircled objects = seed/input nodes; arrows = interactions; green arrows = activation; red arrows = inhibition; grey arrows = unspecified interaction; solid red circles = upregulation; solid blue circles = downregulation. Darker red represents higher upregulation of gene expression (higher log_2_FC); darker blue represents lower downregulation of gene expression (lower log_2_FC).

In order to gain a better understanding of the CMap compound hits, we created functional networks that connected drug targets of the compound hits shown in Table 3. Our drug target list consisted of 62 MetaCore^TM^ network objects considered as network seeds. We used the Analyze Network algorithm in MetaCore^TM^, restricting the network nodes to 50 and using canonical pathways. Our analysis resulted in 18 functional networks connecting drug targets of CMap compound hits (Supplementary Table S3). The top-scoring network (Figure 5) highlighted a potential role for androgen signaling and the upregulation protein kinase C (PKC) on calcium ions (Ca^2+^) homeostasis. The effects of androgen receptor signaling on PKC have been suggested to play a fundamental role in regulating cardiac contractility and Ca^2+^ handling in myocytes [136]. Testosterone induces an increase in the intracellular Ca^2+^ level by a nongenomic mechanism in cultured rat cardiac myocytes [137]. PKC activation of ERK may involve the modulation of intracellular calcium (Ca^2+^) concentration. PKC is upregulated on day 22 (the day after receiving the second dose) in response to BNT162b2. C-C chemokine receptors type 1 (CCR1) and type 2 (CCR2), both upregulated in response to BNT162b2, are known to transduce signals by increasing intracellular calcium levels. The magnitude of the calcium mobilization response can be potentially dependent on both intra- and extracellular stores of calcium [138]. CCR1 and CCR2 use a PLC signaling pathway to activate the same ER calcium store. This network also shows that the androgen receptor is a regularity hub which activates PKC-alpha. Hence, this network is more likely to get perturbed in males than in females in case of increased activation of androgen receptors in response to binding with androgens. These results are consistent with earlier research indicating that testosterone induces intracellular Ca^2+^ increases in cardiac myocytes by increasing the mRNA expression of critical Ca^2+^-handling proteins, resulting in sex-related differences in cardiac function [139]. It is well established that there are male–female differences in intracellular Ca^2+^ release and contraction in ventricular myocytes arising from effects of sex steroid hormones on processes involved in intracellular Ca^2+^ homeostasis [140].

### 2.5. Post-Vaccine Side Effects Most Frequently Reported in VAERS

We explored the VAERS database in order to check whether the most frequently reported adverse events can be explained or predicted based on the systems biology effects highlighted in this study. We found that as of 3 December 2021, for BNT162b2 and mRNA-1273 there were 1,304,092 (for 305,266 total events) and 1,325,436 (for 315,308 total events) adverse events reported in the VAERS database [141], respectively. However, the total number of COVID-19 vaccine doses administered in the United States as of 3 December 2021, was as follows: 270.95 million doses of BNT162b2, 178.15 million doses of mRNA-1273, and 16.77 million doses of Janssen’s [142]. It should be noted that VAERS reports adverse events for individuals vaccinated in the United States only. According to the Center for Disease Control (CDC), BNT162b2 constituted 59% of the total COVID-19 vaccines administered in the United States followed by mRNA-1273 (36%) and Janssen (3.24%). Other vaccines constituted less than 2% of the total administered vaccine doses [143].

**Figure 6:**
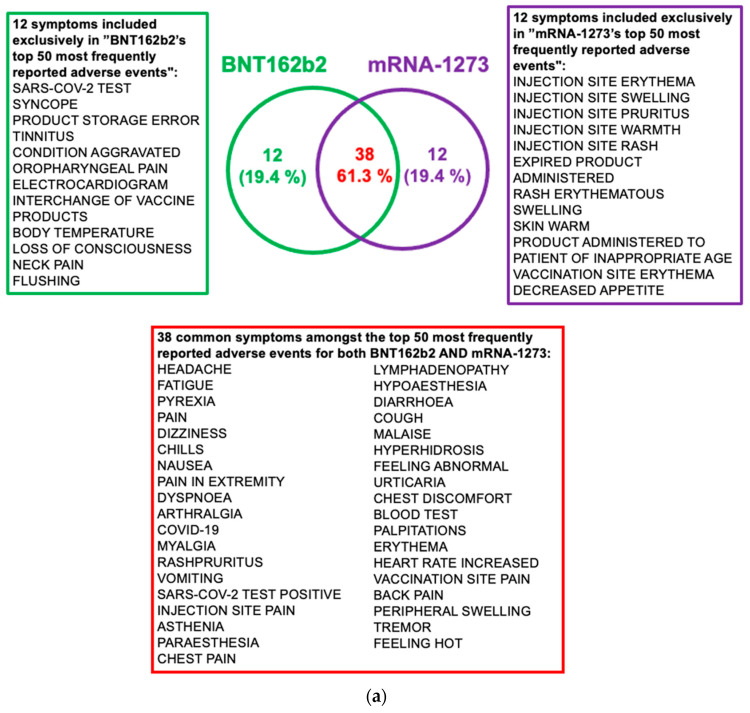

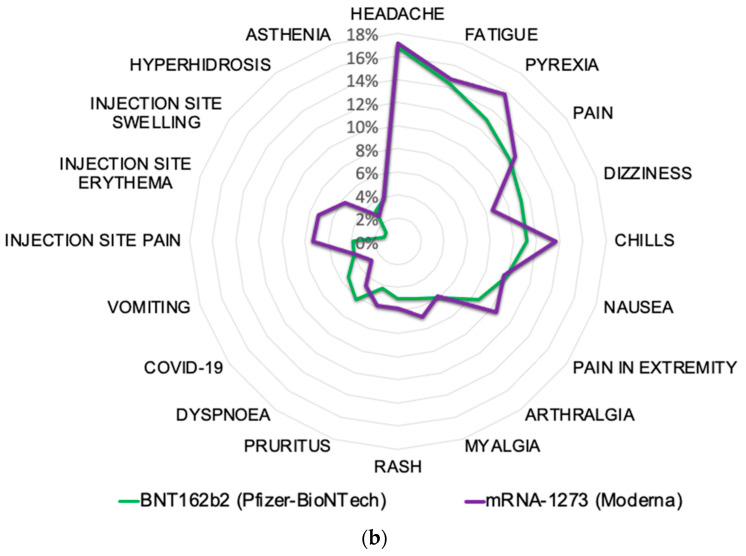
Vaccine adverse events reported for BNT162b2 and mRNA-1273. (**a**) Top 50 most frequent adverse event symptoms reported in VAERS after receiving BNT162b2 and mRNA-1273 COVID-19 vaccines. (**b**) Radar chart to compare the top 15 most frequent adverse events for each vaccine. VAERS reports processed as of 11 December 2021 are considered.

Our search in the VAERS database indicated that the COVID-19 mRNA vaccines BNT162b2 and mRNA-1273 shared 62.4% of the adverse event symptoms. There were 8812 and 8234 adverse event symptoms reported for BNT162b2 and mRNA-1273 vaccines, respectively. The 50 top adverse events for BNT162b2 and mRNA-1273 are shown in Figure 6a, and a comparison of the top 15 adverse event symptoms for the two vaccines is shown in Figure 6b. The complete lists of adverse events are provided in Supplementary Table S4. Headache was the top adverse event symptom reported for the two vaccines. None of the top 15 most frequently reported adverse events was overly concerning or alarming. Interestingly, COVID-19 was the eleventh most frequent adverse event reported for BNT162b2 constituting 5.26% of all adverse events reported for this vaccine. For mRNA-1273, COVID-19 was ranked as the 28th reported adverse event and constituted 2.84% of all adverse events. Based on these numbers, COVID-19 was more frequently reported after receiving BNT162b2 rather than Moderna’s vaccine. However, this result may have been caused by an independent event of contracting SARS-CoV-2 before the vaccine’s immune protection was attained (i.e., before taking the second vaccine dose or right after taking the second dose). In fact, the biological processes which lead to symptomatic COVID-19 are partly mediated by inducing the secretion of pro-inflammatory cytokines which then recruit immune cells to the infected tissues, such as monocytes and macrophages that further increase the secretion of inflammatory cytokines in response to vaccination with BNT162b2. This could imply that individuals who are exposed to the virus around vaccination time are more likely to develop symptomatic COVID-19, urging them to get tested for COVID-19. This can be explained by the overproduction of inflammatory cytokines by both concurrent infection and vaccination.

Another piece of supporting evidence explaining why COVID-19 is developed in 5.26% of people with adverse reactions came from our single-cell transcriptional data analysis, which indicated that several members of the transmembrane protease serine subfamily (TMPRSS) are overexpressed on days 21 and 22 (see Methods for details). TMPRSS proteins have key roles in human hemostasis as well as in promoting certain pathologies, including several types of cancer, and in facilitating the entrance of respiratory viruses into human cells. TMPRSS2 and TMPRSS4 are known to activate the spike protein of the SARS-CoV-2 virus. In fact, TMPRSS4, TMPRSS6, TMPRSS7, and TMPRSS9 were overexpressed in response to second vaccine dose of BNT162b2. This may indicate that BNT162b2 effects on TMPRSS expression may facilitate infection with SARS-CoV-2 if the vaccinated individual contracted the virus. However, it remains unknown whether mRNA-1273 affects the expression of the TMPRSS proteases similarly. We should keep in mind that while COVID-19 is characterized by a decreased immune response and an increased inflammatory response, leading to common vascular pathologies, mRNA vaccines stimulate the immune processes that help fight off SARS-CoV-2, induce antibody production, and induce inflammatory responses which could lead to rare vascular pathologies.

## 3. Discussion

The application of systems biology methods for analyzing the pharmacological responses of vaccines can bolster our understanding of post-vaccine health effects. It can also provide testable hypotheses coupled with mechanistic insights regarding post-vaccine adverse events. Herein, we applied a systems biology workflow for studying vaccine side effects [69,71,144,145]. We used multiple transcriptional gene signatures to assess the pharmacological effects of mRNA vaccines.

Our findings are relevant to the general public, clinicians, regulatory agencies, and policy makers. Our results indicate that BNT162b2 induces a strong immune response in vaccinated individuals on day 22 (i.e., one day after receiving the second, booster dose), as compared to the immune responses evaluated by gene expression on days 1, 2, 21, 23 and 28. The top enriched biological pathways are immune response pathways, which are required for the adequate performance of the vaccines in triggering an adaptive immune response. Enrichment results in process networks highlight a major role of inflammatory and innate-immune processes related to IFN-gamma, interleukins, and protein C signaling. This indicates that the vaccine does its job of stimulating the immune response, however, some of the stimulated innate-immune processes, such INF-gamma signaling, can trigger inflammatory pathways and lead to post-vaccine adverse side effects. Results also highlight the involvement of male and female hormones in some of these inflammatory processes.

High-confidence disease enrichment results (Figure 3) highlight the following diseases associated with genes implicated in transcriptional effects of BNT162b2: xerostomia, rheumatic diseases, immune system diseases, viral diseases, systemic lupus erythematosus, connective tissue disease, autoimmune diseases, Sjogren’s syndrome, arthritis, musculoskeletal disease, lacrimal apparatus diseases, and joint diseases, in addition to skin and connective tissue diseases. These results should be viewed as testable hypotheses for the presence of putative functional links between the BNT162b2 vaccine and the prioritized diseases, and should not be considered as a proof that the vaccine will cause or possibly alienate these ailments. Interestingly, vascular and cardiovascular diseases, which are known risk factors in COVID-19 patients [146], were indeed among the top 50 enriched diseases by DEGs prioritized from single-cell transcriptomics data of BNT162b2. This is a significant result in the context of the recently identified functional link between mRNA COVID-19 vaccines and myocarditis [57].

We also found that BNT162b2 induces immune responses similar to lipopolysaccharide (LPS)-induced platelet activation. In fact, platelets are known to play a significant role in hemostasis and host-defense mechanisms [147,148], in addition to moderating the communication between T- and B-cells, which is crucial for acquired immunity [149]. Platelets can recognize pathogens and recruit host-defense peptides such as platelet factor 4 (PF4), a key platelet-derived CXC chemokine, to promote leukocyte reactions in the immune system [144,145]. Perversely, platelet activation is also a key component in developing thrombosis [150]. Clinical studies have reported on rare, but critical, post-vaccine thrombotic adverse events such as vaccine-induced immune thrombotic thrombocytopenia (VITT) following adenovirus-vector vaccines [145,151] and, in rare cases, mRNA vaccines [38,152,153], implicating anti-platelet factor 4 antibody [154,155,156,157]. We identified 1626 (0.53%) and 1133 (0.36%) post vaccine thrombosis adverse events reported in VAERS after receiving BNT162b2 and mRNA-1273 vaccines, respectively. Platelets and leucocytes form immune thrombotic complexes as a sign of intravascular coagulation in COVID-19 [158,159,160].

Next, we queried the CMap database [80,161,162,163], using three transcriptional gene signatures (QGS1–QGS3) representative of the BNT162b2 vaccine in order to identify small molecule compounds that produce similar transcriptional effects to BNT162b2. ATPase inhibitors were highly enriched in CMap positive connections that regulate gene transcription in a similar manner to BNT162b2, including five high confidence hits (digitoxigenin, ouabain, sarmentogenin, digitoxin, and digoxin), three medium confidence hits (proscillaridin, bufalin, and cinobufagin), and one low confidence hit (xanthohumol). The ability of this approach to identify ATPase inhibitors as mimics of BNT162b2 effects on gene transcription serves as a strong validation for the presented approach. ATPase inhibitors and mRNA vaccines are both linked to heart disease. We previously used this approach to explain the link between mRNA vaccines and post-vaccine myocarditis and pericarditis in male adolescents and male young adults [54,71,149].

Other high-confidence, small-molecule compounds bearing transcriptional similarity to BNT162b2 were the protein synthesis inhibitors such as cycloheximide, homoharringtonine, emetine, and cephaeline. All these compounds are known for their ability to modulate immune responses through stimulating NLRP3 inflammasome and IL-1β receptor signaling [164]. Inflammasomes can sense pathogens via pathogen-associated molecular patterns (PAMP) and damage-associated molecular patterns (DAMPs), and can launch innate immune responses to fight off the intruding pathogens. Therefore, they were considered as important targets for adjuvants in vaccine design [165]. Cationic LNPs, similar to those used in mRNA vaccines, have been shown to work as adjuvants in this regard; they can activate toll-like receptor 2 and NLRP3 inflammasome pathways [166]. This result indicates that mRNA COVID-19 vaccines can act as immunogens by encoding for the SARS-CoV-2 spike protein, and also as powerful adjuvants through their LNP content or per the intrinsic immunostimulatory properties of non-modified RNA, such as that used by CureVac [167].

Finally, we mined all VAERS vaccine adverse event reports for mRNA COVID-19 vaccines BNT162b2 and mRNA-1273. Our results indicate that the most frequently encountered post-COVID-19 vaccine adverse events were minor and short-lived. Additionally, when we analyzed the most frequent adverse effects in the context of disruption in calcium homeostasis, we found that many side effects including headache, fatigue, pyrexia, chills, pain, dizziness, nausea, myalgia, inflammation, seizures, myopathies, etc., are linked to a dysregulation of calcium homeostasis according to chemical-disease search results from the Comparative Toxicogenomics Databases (CTD) [168] as of January 2022. All results are provided in Supplementary Table S5. We also found evidence that interference with the regulation of the intracellular calcium levels in cardiomyocytes can affect the myocardial contractility leading to the development of cardiomyopathy and myocarditis [169]. Nothing was overly concerning in the top 15 adverse events reported in Figure 6, except the observation regarding COVID-19 being among top 15 most frequently reported post-vaccine adverse events. In fact, COVID-19 ranked number 11 of the most frequent adverse event for BNT162b2 and 37 for mRNA-1273. It should be considered alarming that a vaccine may result in immune dysregulation that makes some individuals vulnerable to the disease against which it have been developed, so this observation deserves further scrutiny on the part of vaccine manufacturers and CDC.

We should be cognizant that mRNA vaccines do not contain live virus and thus carry no risk of causing COVID-19 disease in the vaccinated individual without being exposed to the live virus. However, our results indicate that the vaccine may put some people at higher risk of immune dysregulation if infection with the live virus happened right after vaccination and before the person is fully protected by antibodies produced in response to the vaccine. This result is supported by experimental evidence from the transcriptomics data (Supplementary Table S1), coupled with the computational evidence from the enrichment analysis (Figure 3 and Figure 4a,b) and backed by recent evidence from the biomedical literature indicating immune dysregulation involving innate immune pathways and NF-κB signaling following SARS-CoV-2 mRNA vaccinations [62,170,171]. Nevertheless, these results could be confounded by many factors including the effects of host genetic background on the susceptibility to SARS-CoV-2 infection and COVID-19 outcome. For example, variants in cytokine genes and tumor necrosis factor (TNF) affect COVID-19 susceptibility, severity, and complications [172,173]. Other variants in ACE2 and TMPRSS2 affect the expression of the receptors related to COVID-19 and have been associated with disease susceptibility and risk factors [172,173]. Probably, the chances of contracting COVID-19 increase during active epidemic waves for both vaccinated and unvaccinated individuals due to increased viral transmission rates. It would be best if vaccine-adverse event reporting systems clearly indicate if the vaccine was taken during or before an epidemic wave.

Focusing on post-vaccine myocarditis, an adverse event which has recently been linked to both adenovirus and mRNA COVID-19 vaccines, we identified 1365 (0.45%) myocarditis and 871 (0.29%) pericarditis adverse events reported for BNT162b2, and 687 (0.22%) myocarditis as well as 536 (0.16%) pericarditis adverse events reported for mRNA-1273. In fact, a recent population study showed that the risk of developing post-vaccine cardiac events is still small and confined to the 7-day period following vaccination [174]. Additionally, most vaccine-associated myocarditis events have been mild and self-limiting. Contrariwise, the lifetime risk of morbidity and mortality following SARS-CoV-2 infection is substantial [174].

Another inflammatory condition, Sjogren’s syndrome, which is a chronic inflammatory and autoimmune disease where the salivary and lacrimal glands undergo progressive destruction by lymphocytes and plasma cells resulting in decreased production of saliva and tears [77], has been predicted from the enrichment analysis and validated by post-vaccine adverse event reporting in VAERS. There were 42 Sjogren’s syndrome events (0.01%) for BNT162b2 and 21 events (0.01%) for mRNA-1273.

It should be noted that vaccine-induced gene expression perturbations were insignificant on day 28 (no DEGs passed the filtering criteria of log_2_ fold change ≥ 2 and FDR ≤ 0.05). This confirms that vaccine effects on gene expression are temporary and short-term. This finding supports previous claims that mRNA vaccines are safe for the general population. Additionally, world-data has shown that the suggested adverse effects of BNT162b2 are largely non-concerning and lead to minor side effects. However, our network analysis results indicate that aberrant androgen receptor signaling, resulting from certain genetic mutations in Ca^2+^ homeostasis proteins, or driven by increased testosterone levels in males during puberty, may increase the potential for developing adverse events related to abnormalities in Ca^2+^ homeostasis.

Our integrative informatics workflow has several advantages over relying solely on enrichment analyses for adverse event prediction. First, it integrates hypotheses derived independently from the enrichment and CMap analyze to increase the confidence in resulting computational hypotheses. Additionally, the CMap is considered a unique chemogenomics databases capable of connecting genes, drugs, and diseases without requiring a detailed mechanism of action or knowledge of drug targets a priori. In fact, the CMap can easily identify polypharmacologic drugs (or vaccines) from their gene expression profiles. This permits the prediction of drug (or vaccine) adverse events based on similarities with the gene expression profiles of known, well-studied drugs. Cross-examining prioritized hypotheses with vaccine adverse event reports included in the VAERS database serves as a validation step for the prioritized hypotheses. Ultimately, our informatics workflow is capable of filtering high-confidence hypotheses which have higher chances of succeeding in experimental testing and/or clinical studies.

However, there are some limitations to this study, especially due to the scarcity of public transcriptomics datasets for COVID-19 vaccines. Ideally, we would like to study as many transcriptional profiles as possible. Nevertheless, there were only two transcriptomics data sets for BNT162b2 in humans we have identified for this research project. Similar analyses can be carried out to assess other vaccines as transcriptional data in response to treatment with other vaccines becomes available.

In conclusion, our results highlight the important role of mRNA vaccines as potent immunogens leading to the activation of immune-signaling pathways that are essential for mounting a proper immune response in vaccinated individuals. Our analysis has shown a relatively acceptable overall adverse event profile for mRNA vaccines. The most alarming side effects identified by our methods were related to cardiac/cardiovascular events despite being reported as low-frequency adverse events. Our workflow can be used for acquiring essential knowledge for enhancing vaccines and managing, or preventing, post-vaccine adverse events. Potentially, this analysis could lead to personalized recommendations using patient-specific gene signatures, especially if deeper vaccine-genomics studies are conducted.

## 4. Material and Methods

### 4.1. Integrative Informatics Workflow

We developed an informatics workflow (Figure 1), based on the methods developed by Hajjo et al. [54,69,71,148,149], to interrogate the network pharmacology of mRNA vaccines and derive testable mechanistic hypotheses to predict the short-term and long-term side effects of vaccines. These methods rely on developing drug discovery and or network biology hypotheses from different data types and by applying diverse computational methods. First, we started by preparing consensus gene signatures representative of the vaccine’s effects on gene transcription. Next, we queried MetaCore^TM^ [77] and the Connectivity Map of the Broad Institute [175] to prioritize enriched biological processes and compounds with similar effects on gene expression, respectively. Finally, we searched the VAERS database for all adverse events reported for any COVID-19 vaccine included in VAERS. All reports were grouped according to vaccine symptoms and vaccine names. The detailed study workflow is shown in Figure 1.

### 4.2. Data Sets

#### 4.2.1. Vaccine Transcriptional Gene Signatures

We searched the gene expression omnibus (GEO) for transcriptional studies performed in response to treatment with mRNA vaccines and we were able to identify two datasets only; both were for the BNT162b2 vaccine. The first dataset, GSE169159, was a whole transcriptomics dataset in response to treatment with BNT162b2. This dataset was used to generate three query gene lists: GS1, GS2, QGS1, and QGS2. The second dataset, GSE171964 is a single-cell transcriptomics dataset in response to treatment with BNT162b2. This dataset was used to generate query gene lists GS3 and QGS3. Gene signatures GS1–GS3 are provided in Supplementary Table S1, and query gene signatures for the CMap, QGS1–QGS3, are provided in Supplementary Table S6. We analyzed the whole and single-cell transcriptional effects on days 1, 7, 21, 22, 23, and 28. The DEGs in the selected gene signatures passed the DEG filtering thresholds of log_2_ fold change (log_2_FC) of ≥2 or ≤−2, and false discovery rate (FDR) ≤ 0.05. Priming vaccine doses (i.e., first doses) were given to all participant human subjects on day 1. Booster vaccine doses (i.e., second doses) were given on day 21. Gene expression on day 1 prior to receiving the vaccine was considered as the baseline for all differential gene expression comparisons analyzed in this work.

#### 4.2.2. Vaccine Adverse Events Data Set

Raw data files were downloaded as comma-separated value (CSV) files from the CDC website [176]. The CDC WONDER online search tool was used to mine VAERS [177]. We downloaded the 2021 VAERS public data set, which contained all vaccine side reaction reports for COVID-19 vaccines. The COVID-19 vaccines included in the databases were: BNT162b2, mRNA-1273, and Janssen’s.

### 4.3. Databases

#### 4.3.1. VAERS

VAERS [177] was established in 1990 as a national early warning system to detect possible safety problems in U.S.-licensed vaccines. It is co-managed by the U.S. Centers for Disease Control and Prevention (CDC) and the U.S. Food and Drug Administration (FDA). The VAERS data are updated weekly, and include reports from 1990 onwards.

#### 4.3.2. MetaCore™

MetaCore™ version 21.4 build 70,700 [77] from Clarivate Analytics is a database of manually-composed ontologies mapped to canonical pathways and networks. We used this database for purposes of enrichment analysis in pathway maps. Pathway maps in MetaCore™ are defined as subsets of functionally-connected genes to describe a specific cellular process in a specific cellular context. Herein, the enrichment analysis was performed by examining the intersection between a query gene list from transcriptional experiments (i.e., in response to compound treatment with a vaccine) and the prebuilt pathway maps in MetaCore™ using the hypergeometric mean, which takes into account the number of objects in in the query dataset, the number of objects in the intersecting map and the number of objects in the entire database. This assessment returns a *p*-value that tells us the likelihood that the intersection between the gene signature and a particular map is obtained purely by chance. We set the *p*-value threshold at 0.05; rejecting all hypotheses/pathway maps that have enrichment *p*-values higher than 0.05. Additionally, network objects in MetaCore™ are defined as proteins, protein complexes or groups, peptides, RNA species, compounds, EC numbers (function), or reactions. Genes are represented indirectly via the proteins they encode. The same protein in different localizations may be represented by different network objects if its interactions are localization-specific.

#### 4.3.3. Comparative Toxicogenomics Database

The Comparative Toxicogenomics Database (CTD) [168] is a robust, publicly available database that provides manually curated information about chemical–gene/protein interactions as well as chemical–disease and gene–disease relationships.

### 4.4. Network Building

Network building tools in MetaCore™ (version 21.4 build 70700) and Cytoscape (version 3.9.2) were used to build networks. A systematic search, for nearest neighbor (NN) genes/proteins of the upregulated and downregulated genes in BNT162b2′s gene signature, was conducted in Cytoscape version [178] 3.9.0 on Mac OS X 10.16–x86_64, using the STRING protein query application [179]. All retrieved protein–protein interactions (PPIs), including both physical and functional interactions, were retrieved from widely used and reliable databases such as MINT [180], HPRD [181], BIND [182], DIP [183], BioGRID [184], KEGG [185], Reactome [186], EcoCyc [187], NCI-Nature Pathway Interaction Database [188], and Gene Ontology (GO) [189] protein complexes.

Cytoscape was used to generate the network in Figure 2 since we wanted to include the maximal number of direct interactions from diverse databases. MetaCore™ was used to generate the network in Figure 5 since we wanted to focus on manually-curated ontologies that are more capable of providing a mechanistic insight.

### 4.5. OmicSoft Studio

The RNA-seq Analysis Pipeline [190] in OmicSoft Studio, server version 11.2.0.7, was used to determine the differentially expressed genes from the single-cell experiment GSE171964 using gene counts deposited on the Gene Expression Omnibus (GEO) [191]. It was also used for identifying the DEGs from GSE169159 using the gene count data on GEO. We acknowledge the trial access for CLC from Qiagen, which showed great performance for analyzing single-cell data.

## Figures and Tables

**Table 1:** A comparison between COVID-19 mRNA vaccines that are approved, authorized for emergency use, or in late development stage.

Description	Pfizer–BioNTech	Moderna	CureVac
Country	Pfizer (Pfizer, New York, NY, USA)–BioNTech (BioNTech, Mainz, Germany)	Moderna, Cambridge, MA, USA	CureVac, Tübingen, Germany
Vaccine platform [17,18,19,20,21,22,23,24,25]	mRNA: BNT162a1, BNT162b1, BNT162b2, and BNT162c2	mRNA: mRNA-1273	mRNA: CVnCoV
Vaccine genetic material composition (mRNA) [20,26,27,28]	The genetic sequence of full-length spike protein with substituted proline (K986P, V987P) ^a^ N1-methylpseudouridine Codon improvement GC ^b^-enriched content dsRNA ^c^ deletion 5′ CAP1 engineered structure 5′ UTR ^d^: human α-globin RNA with improved Kozak sequence 3′ UTR: AES ^e^ and mtRNR1 ^f^ 3′ UTR motives 110 Poly(A) ^g^ tail with nucleotide-linker (GCAUAUGACU) ^h^	The genetic sequence of full-length spike protein with substituted proline (K986P, V987P) ^a^ N1-methylpseudouridine dsRNA deletion Unrevealed structural components	The genetic sequence of full-length spike protein with substituted proline (K986P, V987P) ^a^ Native nucleotides Modified sequence GC ^b^-enriched content dsRNA ^c^ deletion 5′ CAP1 engineered structure 5′ UTR ^d^: fabricated with Kozak sequence 3′ UTR harboring human α-globin 3′ UTR sequence 64 Poly (A) tail Poly (C) ^i^-rich sequence, succeeded by histone stem loop sequence
LNPs ^j^ composition [4,19,20,21,27,29]	ALC-0315 (synthetic ionizable lipid) = (4-hydroxybutyl) azanediyl)bis(hexane-6,1-diyl)bis (2-hexyldecanoate). ALC-0159 (a synthetic PEGylated lipid) = 2-[(polyethylene glycol)-2000]-N,N-ditetradecyl acetamide. 1,2-distearoyl-sn-glycero-3-phosphocholine. Cholesterol.	SM-102 (synthetic ionizable lipid) PEG2000-DMG = 1-monomethoxypoly ethyleneglycol-2,3-dimyristyl glycerol with PEG2000 ^k^ 1,2-distearoyl-sn-glycero-3 phosphocholine. Cholesterol	Cationic lipid (Synthetic cationic lipid from Acuitas Therapeutics) Phospholipid Cholesterol PEGylated-lipid conjugate excipient.
Molar lipid ratios (%) ionizable cationic lipid: neutral lipid: cholesterol: PEG-ylated lipid [21,27]	46.3:9.4:42.7:1.6	50:10:38.5:1.5	50:10:38.5:1.5
Molar N/P ratios N = nitrogen (ionizable group cationic lipid) P = phosphate (nucleotide group) [21,27]	Vaccine makers evaluated 6 different formulations	Vaccine makers evaluated 6 different formulations	Vaccine makers evaluated 6 different formulations
Buffer [4,19,20,21,29]	Phosphate (PO_4_^−2^) (KH_2_PO_4_, Na_2_HPO_4_·2H_2_O)	Tris (Tromethamine)	NA ^l^
Extra excipients [4,19,20,21,29]	KCl, NaCl, Sucrose, and H_2_O for vaccination	CH_3_COONa, Sucrose, and H_2_O for vaccination	Saline
Dose, dosing regimen, and route of administration [17,18,19,20,21,22,23,24,25]	30 μg (0.3 mL), day 1-day21	100 μg (0.5 mL), day 1-day29	12 μg (NA mL), day 1-day29
Stability condition [18,20,30,31,32]	(−80–60 °C), Up to 6 months	−20 °C, Up to 6 months	≤−60 °C, Up to 3 months
Temperature range (−20–−80 °C)			
Temperature range (−2–−8 °C)	Up to 5 days	Up to 30 days	Up to 3 months
Room temperature	Up to 2 h (mixing with 1.8 mL NaCl expands the span till 6 h)	Up to 12 h	Up to 24 h
Clinical information [2,33,34,35,36,37,38]	BNT162b1 (2417899–75-1), BNT162b2 (2417899-77-3)	mRNA-1273 (2457298-05-2)	CVnCoV ^n^ (2541470-90-8)
CAS ^m^ registry number (RN)			
Clinical trial registration number	NCT04368728; NCT04760132	NCT04470427; NCT04649151; NCT04760132	NCT04449276; ISRCTN73765130 NCT04515147, PER-054-20; NCT04652102, EUCTR2020-003998-22; NCT04652102, EUCTR2020-003998-22; EUCTR2020-004066-19, NCT04674189; NCT04838847; NCT04848467; NCT04860258
Clinical stage	Phase 4	Phase 4	Phase 3
Target protein	Prefusion stabilized (S-2P) ^o^ transmembrane attached whole sequence spike protein	Prefusion stabilized (S-2P) transmembrane attached whole sequence spike protein	Prefusion stabilized (S-2P) transmembrane attached spike protein
Furin cleavage site	Native	Native	Entire S1/S2 ^p^ cleavage domain and transmembrane domain
Real world vaccine effectiveness against original SARS-CoV-2 strain of Wuhan [2,8,39,40,41,42]	64–99%	68–99%	47%
Real world vaccine effectiveness against SARS-CoV-2 variants [2,8,39,40,41,42]	α (B.1.1.7) 65–100% ^q^ (84–100%) ^r^	α (B.1.1.7) 79–100% ^q^ (90–96%) ^r^	α (B.1.1.7) NA ^l^
	β (B.1.351) 75–88% ^q^ (95–100%)^r^	β (B.1.351) 88–96% ^q^ (96–100%) ^r^	β (B.1.351) NA
	γ (P.1) 79–88% ^q^ (95–100%) ^r^	γ (P.1) 79–88% ^q^ (95–100%) ^r^	γ (P.1) NA
	δ (B.1.617.2) 79–88% ^q^ (96%) ^r^	δ (B.1.617.2) NA	δ (B.1.617.2) NA
	o (B.1.1529) NA	o (B.1.1529) NA	o (B.1.1529) NA

^a^ Lysine986Proline and Valine987Proline; ^b^ Guanine-Cytosine; ^c^ double-stranded RNA; 5′ end of eukaryotic mRNA which carries an N(7)-methylguanosine residue linked by a 5′-5′ triphosphate bond with a 2′-O-methyl (i.e., methylating the 2′-OH of the ribose); ^d^ 5′ Untranslated Region; ^e^ homo sapiens amino-terminal enhancer of split; ^f^ Mitochondrially Encoded 12S RRNA; ^g^ Adenine; ^h^ Guanine Cytosine Adenine Uracil Adenine Uracil Guanine Adenine Cytosine Uracil; ^i^ Cytosine; ^j^ lipid nanoparticles; ^k^ Polyethylene glycol; ^l^ not available; ^m^ Chemical Abstracts Service; ^n^ CureVac COVID-19 vaccine; ^o^ two proline substitutions; ^p^ Spike protein Subunit 1/Subunit 2; ^q^ vaccine effectiveness against infection; ^r^ vaccine effectiveness against severe disease.

**Table 2:** Top ten enrichment results for query gene list consisting of DEGs selected by applying log_2_FC ≥ ±5 and FDR ≤ 0.05 thresholds.

#	Pathway Map ^a^	FDR ^b^	#Map Objects ^c^	#Overlapping Objects ^d^	Overlapping Objects
1	Immune response_IFN-alpha/beta signaling via JAK/STAT	2.05 × 10^−1^	62	9	USP18, IP10, CCL2, Apo-2L(TNFSF10), ERAP140, RIG-G, PNPase(old-35), RSAD2, ISG15
2	Immune response_IFN-alpha/beta signaling via MAPKs	9.20 × 10^−7^	73	7	IP10, PL scramblase 1, GCH1, Apo-2L(TNFSF10), RIG-G, RSAD2, ISG15
3	Glomerular injury in Lupus Nephritis	1.37 × 10^−3^	92	5	MDA-5, RIG-I, IP10, CCL2, IFI56
4	Macrophage-induced immunosuppression in the tumor microenvironment	1.37 × 10^−3^	97	5	MSR1, PD-L1, CCL2, PD-L2, IDO1
5	COVID-19: immune dysregulation	1.37 × 10^−3^	100	5	MDA-5, RIG-I, IP10, CCL2, ISG15
6	Macrophage and dendritic cell phenotype shift in cancer	1.37 × 10^−3^	100	5	MSR1, IP10, Apo-2L(TNFSF10), SOCS1, IDO1
7	Immune response_IFN-gamma actions on extracellular matrix and cell differentiation	1.73 × 10^−3^	54	4	OAS2, IP10, GCH1, 2′-5′-oligoadenylate synthetase
8	Vascular endothelial cell damage in SLE	2.79 × 10^−3^	63	4	MSR1, PD-L1, CCL2, PD-L2
9	Immune response_Innate immune response to RNA viral infection	4.07 × 10^−3^	28	3	MDA-5, RIG-I, LGP2
10	Immune response_IFN-gamma actions on blood cells	4.07 × 10^−3^	28	3	PD-L1, PD-L2, SOCS1

^a^ Pathway map in MetaCore™ (a graphic image representing complete biochemical pathways or signaling cascades in a commonly accepted sense. Typically, a map comprises 3–5 MetaCore™ pathways. Maps are assembled into map folders divided into regulatory, metabolic, disease, toxicity, and drug action sections, and thus form an ontology of their own kind); ^b^ false discovery rate; ^c^ total network objects on the corresponding pathway map; ^d^ the number of overlapping network objects from query 1 applying a fold change threshold of 5 and −5 on upregulated and downregulated genes, respectively.

**Table 3:** Small-molecule drugs and chemical compounds that regulate gene expression in a similar manner to BNT162b2 vaccine.

#	Compound	CMap Score ^a^	Description	Confidence ^b^	Immune Effects
1	Cycloheximide	98.31	Protein synthesis inhibitor	High	[82,83,84,85]
2	QL-XII-47	96.48	BTK inhibitor	High	[86,87,88]
3	Homoharringtonine	94.71	Protein synthesis inhibitor	High	[89,90,91]
4	Periplocymarin	94.38	Apoptosis stimulant	High	[92,93]
5	Digitoxigenin	94.11	ATPase inhibitor	High	[94,95]
6	Emetine	94.05	Protein synthesis inhibitor	High	[96,97]
7	Ouabain	93.62	ATPase inhibitor	High	[98,99]
8	Cephaeline	92.26	Protein synthesis inhibitor	High	[100]
9	Clofarabine	92.11	Ribonucleoside reductase inhibitor	High	[101]
10	Sarmentogenin	91.66	ATPase inhibitor	High	[102]
11	Digitoxin	91.34	ATPase inhibitor	High	[103]
12	Isoliquiritigenin	90.85	Guanylate cyclase activator	High	[104]
13	Digoxin	90.38	ATPase inhibitor	High	[105,106]
14	Tyrphostin-AG-126	98.84	ERK1/2 phosphorylation inhibitor	Intermediate	[107]
15	Amonafide	98.73	Topoisomerase inhibitor	Intermediate	[108]
16	Diphenoxylate	98.50	Opioid receptor agonist	Intermediate	[109]
17	Verrucarin-a	98.38	Protein synthesis inhibitor	Intermediate	[110]
18	Withaferin-a	97.96	IKK inhibitor	Intermediate	[111]
19	Dapsone	96.32	Bacterial antifolate	Intermediate	[112]
20	Teniposide	95.99	Topoisomerase inhibitor	Intermediate	[113,114]
21	Ziprasidone	95.86	Dopamine receptor antagonist	Intermediate	[115]
22	RO-90-7501	95.80	Beta amyloid inhibitor	Intermediate	[116]
23	XMD-1150	95.38	Leucine rich repeat kinase inhibitor	Intermediate	[117]
24	Ingenol	94.47	PKC activator	Intermediate	[118,119]
25	XMD-892	93.85	MAP kinase inhibitor	Intermediate	[117]
26	Anisomycin	93.69	DNA synthesis inhibitor	Intermediate	[120]
27	Proscillaridin	91.37	ATPase inhibitor	Intermediate	[121]
28	Azacitidine	91.29	DNA methyltransferase inhibitor	Intermediate	[122]
29	4-hydroxy-2-nonenal	90.47	Cytotoxic lipid peroxidation product	Intermediate	[123]
30	Dubinidine	90.37	Anti-epileptic	Intermediate	[124,125]
31	BNTX	89.95	Opioid receptor antagonist	Intermediate	[126]
32	Narciclasine	89.85	Coflilin signaling pathway activator	Intermediate	[127]
33	Mitomycin-c	88.45	DNA alkylating agent	Intermediate	[17]
34	Bufalin	87.17	ATPase inhibitor	Intermediate *	[128]
35	Cinobufagin	86.79	ATPase inhibitor	Intermediate *	[19]
36	Brefeldin-a	86.54	Protein synthesis inhibitor	Intermediate *	[23]
37	Pyrvinium-pamoate	78.80	AKT inhibitor	Low	[24]
38	Liothyronine	72.93	Thyroid hormone stimulant	Low	[25]
39	CD-437	70.27	Retinoid receptor agonist	Low	[22,129]
40	Terreic-acid	66.04	BTK inhibitor	Low *	[130]
41	Minaprine	63.46	Serotonin reuptake inhibitor	Low	[131]
42	Cucurbitacin-i	61.47	JAK inhibitor	Low	[132]
43	Xanthohumol	60.13	ATPase inhibitor	Low *	[133]
44	Benzo(a)pyrene	54.66	Carcinogen	Low	[134,135]

^a^ CMap score, representing the level of similarity between transcriptional effects induced by BNT162b2 vaccine and each of the compounds, only the highest score is shown if two query gene signatures shared the same hit; ^b^ confidence is divided into three levels: high confidence means that the compound has a positive CMap score with two query gene signatures representing the vaccine, and has at least one CMap score ≥ 90; medium confidence means the compound has a positive CMap score score ≥ 90 with one query gene signature only, or it was a hit resulting from two query gene signatures, and has a positive CMap score ≥ 80 with at least one query gene signature; low confidence means the compound has a positive CMap score < 80 with all query gene signatures. * = The compound belongs to a target family of a higher confidence compound hit (which gives more confidence in such hits despite their lower CMap scores).

## Data Availability

Single-cell data files used for the enrichment and CMap analyses are available on GitHub (https://github.com/rhajjo/mRNA-Vaccines, accessed on 20 June 2022).

## Supplementary Materials

The following supporting information can be downloaded at: https://www.mdpi.com/article/10.3390/pathogens11070743/s1. The following are available online at GitHub: Supplementary Table S1, transcriptional signatures GS1–GS5. Supplementary Table S2, top 50 enrichment results for BNT162b2 gene signatures in pathway maps, process networks, diseases, and GO processes. Supplementary Table S3, network list for drug targets of CMap compound hits. Supplementary Table S4, post-vaccine adverse events in response to BNT162b2, mRNA-1273, and Janssen’s. Supplementary Table S5, calcium–disease connections mined from the CTD database. Supplementary Table S6, query gene lists for the CMap. Supplementary Text File S1, example of a log file for analyzing whole transcriptomics data. Supplementary Text File S2, example of a log file for analyzing single-cell transcriptomics data from the counts data file.

## Abbreviations

ACE2	Angiotensin-converting enzyme 2
Ang II	Angiopoietin II
BTK	Bruton’s Tyrosine Kinase
Calcium	Ca^2+^
CDC	Centers for Disease Control and Prevention
CMap	Connectivity map
COVID-19	Coronavirus disease of 2019
CTD	Comparative toxicogenomics database
DEG	Differentially expressed gene
FC	Fold change
FDA	Food and Drug Administration
FDR	False discovery rate
GEO	Gene expression omnibus
GS	Gene signature
INF-gamma	Interferon gamma
LNP	Lipid nanoparticle
mRNA	Messenger ribonucleic acid
SARS-CoV-2	Severe acute respiratory syndrome coronavirus 2
TNF	Tumor necrosis factor
VAERS	Vaccine Adverse Event Reporting System
